# Retarded PDI diffusion and a reductive shift in poise of the calcium depleted endoplasmic reticulum

**DOI:** 10.1186/s12915-014-0112-2

**Published:** 2015-01-10

**Authors:** Edward Avezov, Tasuku Konno, Alisa Zyryanova, Weiyue Chen, Romain Laine, Ana Crespillo-Casado, Eduardo Pinho Melo, Ryo Ushioda, Kazuhiro Nagata, Clemens F Kaminski, Heather P Harding, David Ron

**Affiliations:** University of Cambridge, Cambridge Institute for Medical Research, Biomedical Campus, Wellcome Trust/MRC Building, Hills Road, Cambridge, CB2 0XY United Kingdom; Department of Chemical Engineering and Biotechnology, University of Cambridge, Cambridge, CB2 3RA UK; Center for Biomedical Research, Universidade do Algarve, Faro, Portugal; Faculty of Life Sciences, Kyoto Sangyo University, Kita-Ku, Kyoto-City 603-8555 Japan

**Keywords:** Fluorescence lifetime imaging, Protein disulfide isomerase, Calreticulin, Endoplasmic reticulum, Redox, Calcium

## Abstract

**Background:**

Endoplasmic reticulum (ER) lumenal protein thiol redox balance resists dramatic variation in unfolded protein load imposed by diverse physiological challenges including compromise in the key upstream oxidases. Lumenal calcium depletion, incurred during normal cell signaling, stands out as a notable exception to this resilience, promoting a rapid and reversible shift towards a more reducing poise. Calcium depletion induced ER redox alterations are relevant to physiological conditions associated with calcium signaling, such as the response of pancreatic cells to secretagogues and neuronal activity. The core components of the ER redox machinery are well characterized; however, the molecular basis for the calcium-depletion induced shift in redox balance is presently obscure.

**Results:**

*In vitro*, the core machinery for generating disulfides, consisting of ERO1 and the oxidizing protein disulfide isomerase, PDI1A, was indifferent to variation in calcium concentration within the physiological range. However, ER calcium depletion *in vivo* led to a selective 2.5-fold decline in PDI1A mobility, whereas the mobility of the reducing PDI family member, ERdj5 was unaffected. *In vivo*, fluorescence resonance energy transfer measurements revealed that declining PDI1A mobility correlated with formation of a complex with the abundant ER chaperone calreticulin, whose mobility was also inhibited by calcium depletion and the calcium depletion-mediated reductive shift was attenuated in cells lacking calreticulin. Measurements with purified proteins confirmed that the PDI1A-calreticulin complex dissociated as Ca^2+^ concentrations approached those normally found in the ER lumen ([Ca^2+^]K_0.5max_ = 190 μM).

**Conclusions:**

Our findings suggest that selective sequestration of PDI1A in a calcium depletion-mediated complex with the abundant chaperone calreticulin attenuates the effective concentration of this major lumenal thiol oxidant, providing a plausible and simple mechanism for the observed shift in ER lumenal redox poise upon physiological calcium depletion.

**Electronic supplementary material:**

The online version of this article (doi:10.1186/s12915-014-0112-2) contains supplementary material, which is available to authorized users.

## Background

Cysteine side chains of cytosolic, nuclear and mitochondrial matrix proteins are maintained in their reduced state by a vigorous reductive pathway effected by a large pool of reduced thioredoxins. By contrast, in the lumen of the endoplasmic reticulum (ER) dithiol oxidation and disulfide reduction co-exist. These competing reactions define the redox poise of the ER, which is dominated by the twenty or so members of the protein disulfide isomerase family, the ER counterparts of the thioredoxins (reviewed in [1]).

Oxidizing members of the protein disulfide isomerase (PDI) family, exemplified by PDI1A [2], gain disulfides from upstream oxidants, such as ERO1, PRDX4 and VKOR1C and donate them to reduced ER client proteins (reviewed in [3]). Reducing members of the PDI family, exemplified by ERdj5, [4,5] attack misplaced disulfides on ER clients, and the acquired disulfides are reduced by yet-to-be discovered mechanism(s), re-cycling the reducing PDI to its active state.

Changes to the redox poise of the ER can be tracked by suitably tuned sensors; these are proteins with a metastable disulfide that is kinetically-coupled to the lumenal PDIs and whose redox status influences a measureable outcome, typically fluorescent properties [6,7]. Applying such sensors has revealed that cultured mammalian cells effectively defend their redox poise: neither imposition of unfolded protein load (so-called ER stress), nor manipulations that compromise the upstream oxidases, affect the steady-state disposition of ER localized redox sensors [8,9].

Manipulations that affect ER calcium stores present an intriguing contrast to this theme of stability of ER redox poise. Inhibition of calcium re-uptake into the ER, by targeting the smooth endoplasmic reticulum calcium ATPase (the SERCA) pump with thapsigargin resulted in a reductive shift in ER thiol redox poise. This reductive shift is robust, as it has been observed with redox probes that have different biochemical properties: both the OxyR-YFP fusion known as HyPer [10], which in the ER is predominantly a thiol redox probe [11], and roGFPiE [9,12] and by measurements that exploit either redox-dependent shifts in excitation profiles [10,12] or changes in fluorescence lifetime [9].

*In vitro* measurements excluded a direct effect of calcium on the redox probe, validating the observed changes as faithful reflections of a reductive shift of the calcium-depleted ER lumen. Furthermore, in a cell culture model of a professional secretory cell - the AR42j pancreatic acinar cell line - physiological mobilization of calcium stores by engagement of the cholecystokinin receptor, which couples to inositol trisphosphate receptor (IP3R) release channels, promoted a similar reductive shift in the ER [9]. The link between calcium release and changes to ER redox thus extends to the physiological range of ER calcium signaling, implying that fluctuations in ER redox accompany activity-dependent oscillations in calcium stores of secretory cells. Here, we report on findings that suggest a molecular mechanism for this pervasive phenomenon.

## Results

### The core machinery for disulfide bond formation is indifferent to changes in calcium concentrations in the physiological range

**Figure 1:**
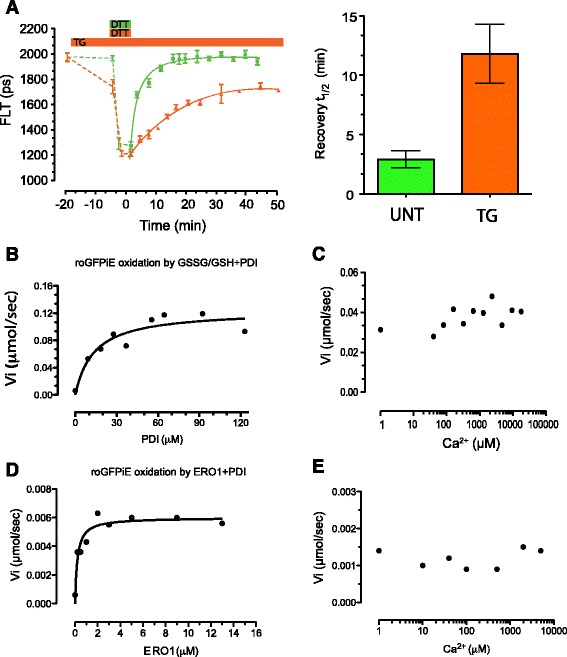
**Calcium insensitivity of the core disulfide oxidative machinery of the ER. (A)** Trace of time-dependent changes in fluorescent lifetime (in picoseconds) of ER-localized roGFPiE preceding and following a dithiothreitol (DTT, 5 mM) pulse and washout in untreated (green) and thapsigargin (TG)-treated (orange) HEK 293 T cells. Each data point represents the mean ± SD of fluorescence lifetime measured in ≥8 cells. Note the shortening of the lifetime and slowness of its recovery following DTT reduction in the thapsigargin-treated group. A comparison of the mean half-time to recovery in the untreated and treated cells is provided in the bar diagram to the right (n >10, *P* <0.05%). **(B)** Plot of the initial velocity of roGFPiE oxidation *in vitro* (measured by the ratio of excitation intensity at 470 nm and 395 nm) in the presence of varying concentration of PDI1A, in a glutathione redox buffer (5:1 GSH/GSSG, 4 mM total, no added Ca^2+^). Kinetic parameters were extracted by fitting the data to a non-linear regression. **(C)** As in ‘B’, with PDI1A held at K_0.5_max (16 μM) and varying concentration of Ca^2+^. **(D)** As above, but in the presence of a saturating concentration of PDI1A (40 μM) and varying concentration of ERO1 (no added Ca^2+^). **(E)** As above, but in the presence of a saturating concentration of PDI1A (40 μM), ERO1 at K_0.5_max (0.5 μM) and varying concentration of Ca^2+^. ER, endoplasmic reticulum; PDI1A, protein disulfide isomerase 1A; SD, standard deviation.

To determine if the previously noted reductive shift in the steady state poise of the ER affected by calcium depletion had a kinetic component, we compared the rate of recovery of the disulfide in reduced roGFPiE following a dithiothreitol (DTT) pulse and its washout in untreated cells and in cells exposed to the ER calcium re-uptake inhibitor thapsigargin. The fluorescent lifetime of the probe declined in thapsigargin-treated HEK 293 T cells, reflecting the reductive shift in the dithiol-disulfide steady state [9]. A DTT reductive pulse further lowered the lifetime (to a similar low baseline in untreated and thapsigargin-treated cells), but its rate of recovery was noticeably slower in the thapsigargin-treated cells: half time to recovery 2.9 ± 0.71 minutes (untreated) and 11.8 ± 2.48 minutes (thapsigargin-treated cells) (Figure 1A).

roGFPiE is kinetically coupled to the major ER oxidoreductase PDI1A and isolated from the small molecule redox buffer glutathione [9]. To determine if the aforementioned delay in oxidation observed *in vivo* reflected a direct effect of calcium concentration on the relevant enzymes, we reconstructed the oxidative cascade linking molecular oxygen to reduced roGFPiE *in vitro*, using purified ERO1 and PDI1A, and established conditions for dependence on the two enzymes. Oxidation of reduced roGFPiE by PDI1A, pre-equilibrated with a glutathione redox buffer, was half-maximal at [PDI1A] K_0.5max_ = 16 μM (Figure 1B), but the oxidation of roGFPiE was unaffected by calcium concentrations over a range of 1 μM to 10 mM (Figure 1C). The concentration-dependence of roGFPiE oxidation on ERO1 (in the presence of saturating concentrations of PDI) was steep, but at ERO1 concentration of 0.5 μM, below the half-maximal concentration (Figure 1D), oxidation of roGFPiE was indifferent to calcium variation over a broad range (Figure 1E).

### PDI1A mobility is retarded by ER calcium depletion *in vivo*

**Figure 2:**
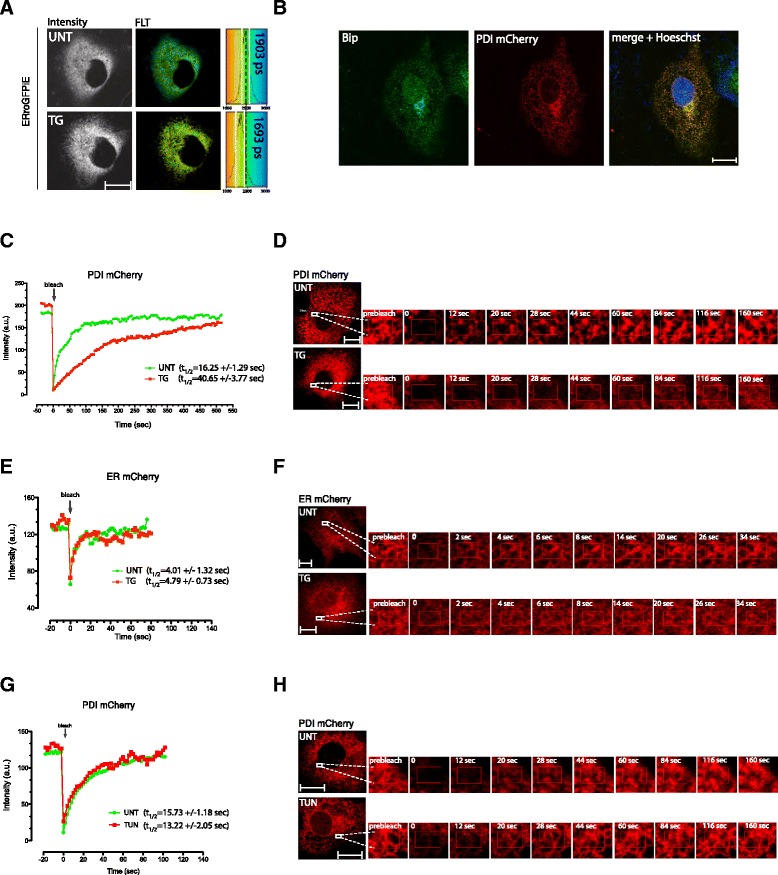
**Impaired PDI1A mobility in the calcium-depleted ER. (A)** Fluorescent lifetime imaging of ER roGFPiE in untreated and thapsigargin-treated COS7 cells. Note the decline in mean lifetime, from 1.903 ns to 1.693 ns, reflecting a reductive shift in the probe. A histogram of the distribution of lifetimes in the cells is provided (right) noting the mean ± SD (vertical white lines) lifetime, on the background of the color code corresponding to the pseudocolored image. A vertical dashed line extending through both panels is provided for alignment of the two measurements. **(B)** Photomicrographs of immunostaining of endogenous BiP (an ER marker, green), PDI1A-mCherry fluorescence (red) and an overlay of the two (yellow) in a COS7 cell transfected with a plasmid encoding an ER-localized PDI1A-mCherry (Pearson’s coefficient of BiP/PDI1A-mCherry co-localization r = 0.89). The purple Hoechst stains the nucleus. **(C)** Trace of time-dependent changes in the fluorescence intensity of PDI1A-mCherry before and after photobleaching a small patch of transfected COS7 cell volume. The green trace is of an untreated cell and the orange of a cell exposed to thapsigargin 10 minutes before data acquisition. **(D)** Photomicrographs of the PDI1A-mCherry fluorescence in the time-dependent series quantified in ‘C’ above. **(E)** As in ‘C’, except that cells were transfected with an expression plasmid encoding ER-localized mCherry. **(F)** As in ‘D’ but from the time series of the ER mCherry-expressing cells. **(G)** As in ‘C’, but cells were treated with the glycosylation inhibitor tunicamycin to promote unfolded protein stress in the ER, without affecting ER Ca^2+^ levels. **(H)** Time series of the sample shown in **(G)**. The mean ± SD of the half-time to recovery of the two samples is provided. The size bar is 20 μm. ER, endoplasmic reticulum; PDI1A, protein disulfide isomerase 1A; standard deviation.

The experiments presented above suggest that the effects of calcium on the ER redox poise observed in cells lack a simple *in vitro* counterpart in terms of the activity of the core oxidative machinery of the ER. To explore features that might be lacking from the simplified *in vitro* system, we compared the mobility of fluorescently-tagged PDI1A in untreated and calcium-depleted cells using fluorescent recovery after photobleaching (FRAP). To obtain suitable spatial resolution of ER imaging we sought to make use of COS7 cells (with superior ER morphology) and firstly confirmed that in these cells, too, thapsigargin-induced ER calcium depletion resulted in a reductive shift of the ER (Figure 2A). An mCherry genetically-encoded fluorescent probe was inserted into PDI1A, N-terminal to the KDEL ER retention signal. When transfected into COS7 cells the fluorescent signal produced a typical ER pattern that colocalized with the endogenous ER chaperone BiP (Figure 2B).

The PDI1A-mCherry fluorescence signal recovered 2.5 times more rapidly in a photobleached patch of ER from untreated cells compared with cells treated with thapsigargin (Figure 2C,D). The recovery of ER localized mCherry fluorescence was unaffected by thapsigargin (Figure 2E,F), as was the recovery of PDI1A-mCherry in cells treated with the glycosylation inhibitor tunicamycin, which induces high levels of ER stress, without affecting calcium (Figure 2G,H and Additional file 1: Figure S1). Importantly, ER calcium depletion retarded PDI1A mobility even in cells exposed to DTT (see Additional file 1: Figure S2), implying that reduced PDI1A is also subject to immobilization, a point we shall return to later.

### PDI1A binds calreticulin in the calcium depleted ER

**Figure 3:**
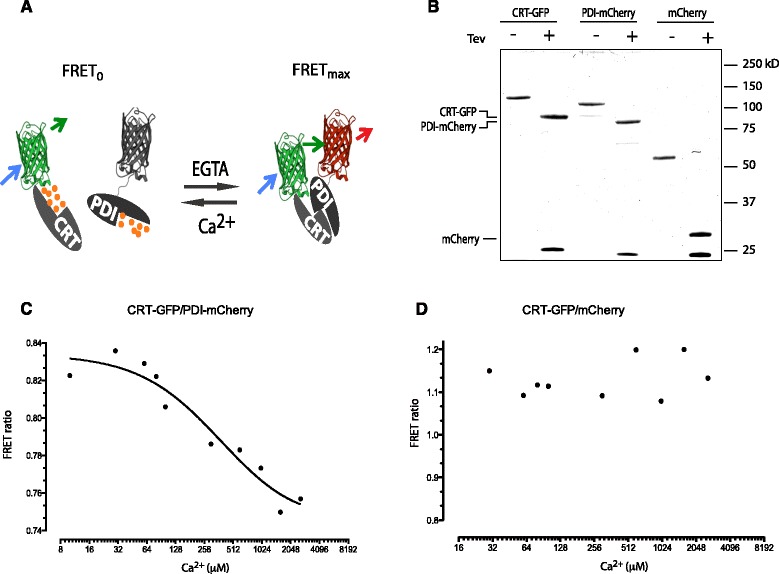
**Calcium concentration-dependent association of PDI1A and calreticulin** ***in vitro*** **. (A)** Schema of the calcium-dependent fluorescence resonance energy transfer (FRET) between a green fluorescent protein tagged calreticulin (CRT-GFP) donor and a monomeric Cherry tagged PDI1A (PDI1A-mCherry) acceptor. **(B)** Photomicrograph of Coomassie-stained fusion proteins, before and after TEV protease cleavage of the GST tag, resolved on a 10% SDS-PAGE. **(C)** Trace of calcium concentration-dependent changes in FRET between the CRT-GFP donor and the PDI1A-mCherry acceptor (both at 12 μM). The half-maximal change in the FRET signal is observed at 190 μM free Ca^2+^. Shown is a typical trace of an experiment reproduced three times. **(D)** As in ‘C’ above, but substituting mCherry for PDI1A-mCherry as the FRET acceptor. PDI1A, protein disulfide isomerase 1A.

The calcium depletion-dependent attenuation in PDI1A’s mobility in the ER was consistent with its incorporation into a complex with other protein(s) that were absent from the *in vitro* reconstituted system described in Figure 1B-E. Michalak and colleagues had previously noted quenching of fluorescently-labeled PDI1A by calreticulin (CRT) upon calcium chelation in an *in vitro* system, suggesting an interaction that could explain our *in vivo* observations [13]. To explore this possibility further, we purified PDI1A-mCherry and CRT-GFP from bacteria and measured the interaction between them by the intensity of the fluorescence resonance energy transfer (FRET) signal, as reflected in acceptor (PDI1A-mCherry)-dependent increase in FRET ratio (Figure 3A,B). The FRET signal decreased progressively with calcium concentration, with a midpoint, [Ca^2+^]_0.5_, of 190 μM (Figure 3C). These [Ca^2+^]-dependent changes in FRET ratio were not observed with mCherry as the acceptor (Figure 3D), attesting to the specificity of the PDI1A-CRT interaction originally observed by Michalak and colleagues.

The CRT tertiary structure is modulated by calcium concentration, with enhanced exposure of hydrophobic side-chains at low calcium concentrations [14]. In keeping with this, we observed CRT precipitation with declining concentration of calcium in the buffer. CRT precipitation was prevented by including PDI1A in the solution (see Additional file 1: Figure S3), supporting the FRET measurements noted above and suggesting a role for the calcium-dependent association of the two proteins in maintaining CRT’s solubility.

**Figure 4:**
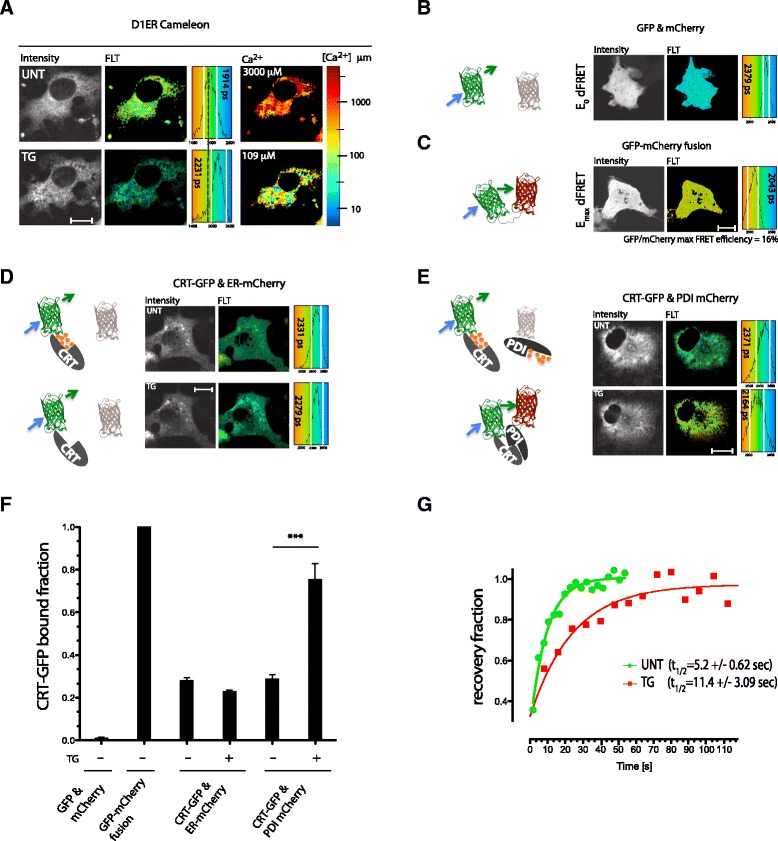
**Calcium depletion-dependent association of PDI1A and calreticulin** ***in vivo*** **. (A)** Donor (cyan fluorescent protein) fluorescence (intensity) and donor lifetime images (FLT) of an ER calcium probe, D1ER Cameleon, in untreated and thapsigargin-treated COS7 cells. A histogram of the distribution of lifetimes in the cells is provided (right) noting the mean ± SD (vertical white lines) lifetime. Also shown is a pseudo-colored image of calcium concentration derived from Cameleon’s lifetime. Note the decline in mean [Ca^2+^]_ER_ from approximately 3 mM to 109 μM in the treated sample. **(B)** Donor (GFP) fluorescent lifetime of GFP transfected alongside mCherry, setting the lower limits of the FRET for this pair. The size bar is 20 μm. **(C)** Donor (GFP) fluorescent lifetime a fusion protein of GFP and mCherry, setting an upper limit of FRET for this pair. **(D)** Donor (GFP) fluorescent lifetime of CRT-GFP fusion protein transfected alongside an ER mCherry, as a FRET acceptor. Where indicated the cells were treated with thapsigargin (TG). **(E)** Donor (GFP) fluorescent lifetime of CRT-GFP fusion protein transfected alongside PDI1A-mCherry, as a FRET acceptor. Where indicated the cells were treated with thapsigargin (TG). **(F)** Bar diagram of fraction of GFP probe bound to mCherry probe derived from the FRET measurements in B-E (mean ± SEM, n >100, *P* <0.001). **(G)** Trace of time-dependent changes in the normalized intensity of CRT-GFP after photobleaching a small patch of transfected COS7 cell volume. The green trace is of untreated sample and the red of cells exposed to thapsigargin 10 minutes before data acquisition. The mean ± SD of the half-time to recovery of the two samples is provided. CRT, calreticulin; ER, endoplasmic reticulum; FRET, fluorescence resonance imaging measurement; SD, standard deviation; SEM, standard error of the mean.

To explore the possible *in vivo* relevance of the PDI1A-CRT interaction we exploited the superior accuracy of fluorescent lifetime imaging (FLIM) measurements to detect FRET [15]. When applied to thapsigargin-treated COS7 cells, a calibrated ER-localized calcium probe (D1ER cameleon, whose FRET signal increases with calcium concentration) [16], measured in FLIM-FRET mode, revealed a decline in [Ca^2+^]_ER_ (reflected in enhanced donor lifetime) into the range at which PDI1A and CRT were observed to interact *in vitro* (Figures 3C and 4A). This finding encouraged us to measure the effects of changes in ER calcium on the interaction between PDI1A and CRT *in vivo*. The dynamic range of *in vivo* measurements of FRET between a GFP donor and an mCherry acceptor were established by expressing GFP and mCherry separately and as a fusion protein in the ER. Fusion of the two led to a drop in donor fluorescence lifetime (from 2.39 ± 0.04 ns to 2.04 ± 0.06 ns) (Figure 4B,C), setting an upper limit for FRET efficiency of this pair. When co-expressed with free mCherry, lifetime of a CRT-GFP donor was unaffected by ER calcium depletion (Figure 4D); however, a reproducible thapsigargin-dependent drop in donor lifetime was observed when a PDI1A-mCherry acceptor was introduced into the ER (Figure 4E,F). FRET with the PDI1A-mCherry acceptor further correlated with a two-fold attenuation in CRT-GFP’s mobility as assessed by FRAP measurements in thapsigargin-treated cells (Figure 4G), further suggesting the formation of a complex between the two proteins in the calcium depleted ER.

**Figure 5:**
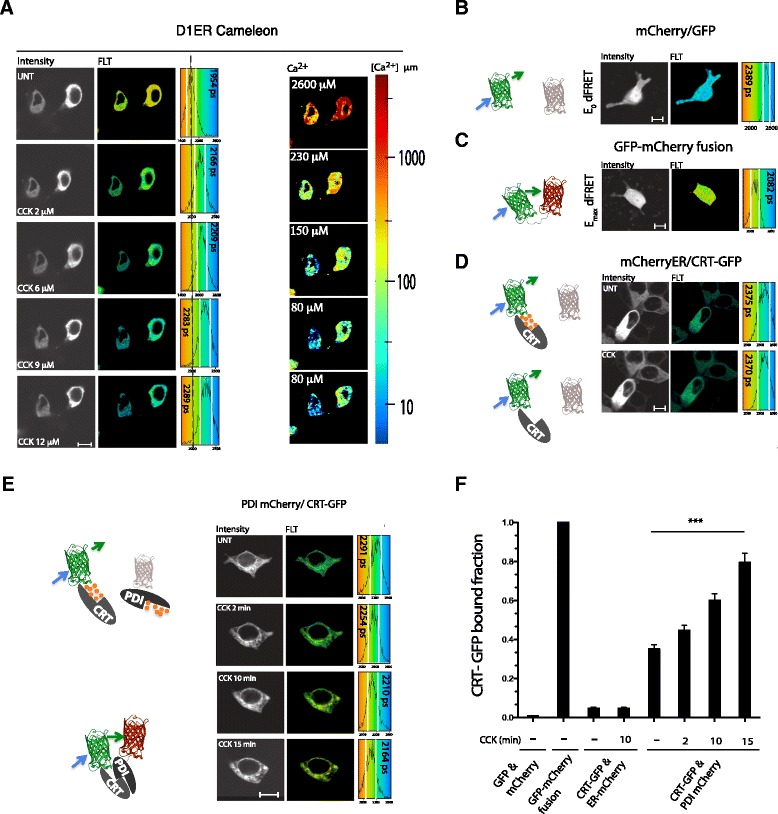
**Association of PDI1A and calreticulin under physiological conditions of calcium depletion. (A)** Donor (cyan fluorescent protein) fluorescent intensity and donor life time images (FLT) of D1ER Cameleon in untreated and cholecystokinin (CCK)-treated (10 minutes) pancreatic acinar AR42j cells. A histogram of the distribution of lifetimes is provided (right) noting the mean ± SD (vertical white lines) lifetime of the cells in the sample. Further to the right is a pseudo-colored image of calcium concentration derived from Cameleon’s life time and notation of the mean of the concentration in each sample. Note the decline in mean [Ca^2+^]_ER_ from 2.6 mM in the untreated to 80 μM in the sample exposed to the highest concentration of CCK. The size bar is 20 μm. **(B)** Donor (GFP) fluorescent lifetime of GFP transfected alongside mCherry, setting the lower limits of the FRET for this pair in AR42j cells. **(C)** Donor (GFP) fluorescent lifetime of a fusion protein of GFP and mCherry, setting an upper limit of FRET for this pair in AR42j cells. **(D)** Donor (GFP) fluorescent lifetime of CRT-GFP fusion protein transfected alongside and ER mCherry, as a FRET acceptor. Where indicated the cells were treated with CCK (10 μM for 15 minutes). **(E)** Donor (GFP) fluorescent lifetime of CRT-GFP fusion protein transfected alongside PDI1A-mCherry, as a FRET acceptor. Samples were treated with CCK (10 μM) for the indicated time. **(F)** Bar diagram of fraction of GFP probe bound to mCherry probe derived from the FRET measurements in B-E (mean ± SEM, n >100, *P* <0.001). The size bar is 20 μm. CRT, calreticulin; FRET, fluorescence resonance energy transfer; PDI1A, protein disulfide isomerase 1A; SD, standard deviation; SEM, standard error of the mean.

To address the potential physiological relevance of the interaction between PDI1A and CRT, we turned to AR42j cells, a pancreatic acinar cell line in which physiological calcium store depletion is coupled to stimulation by the hormone cholecystokinin, resulting in a reductive shift in the ER [9]. Calcium imaging revealed a cholecystokinin dose-dependent dip in [Ca^2+^]_ER_ into the range at which PDI1A and CRT were observed to interact *in vitro* (Figure 5A). Expectedly, GFP to mCherry FRET was readily observed in these cells (Figure 5B,C), as was the lack of an interaction between the CRT-GFP donor and a free mCherry acceptor (Figure 5D). However, conspicuous time-dependent FRET from the CRT-GFP donor to the PDI1A-mCherry acceptor was also observed in cholecystokinin-treated cells (Figure 5E,F), consistent with the formation of a complex between the two proteins upon physiological ER calcium store emptying.

Association of PDI1A with calreticulin was also observed in DTT-treated cells (see Additional file 1: Figure S4), implying that CRT-binding was not restricted to the oxidized form of PDI1A, which is noted for its higher affinity for unfolded proteins [17]. Furthermore, complex formation with CRT did not affect PDI1A’s ability to catalyze the oxidation of reduced roGFPiE *in vitro* (see Additional file 1: Figure S5). These negative results leave altered PDI1A mobility as the notable correlate of calcium depletion.

**Figure 6:**
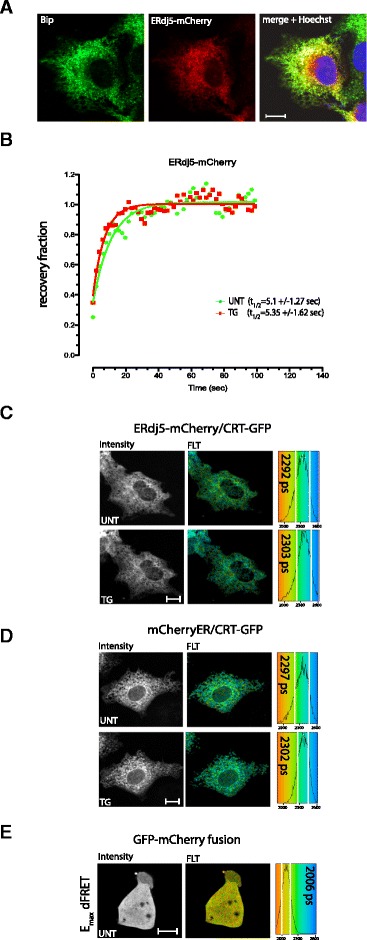
**Mobility of ERdj5, a reducing member of the PDI family, is unaffected by ER calcium depletion. (A)** Photomicrographs of immunostaining of endogenous BiP (an ER marker green), ERdj5-mCherry fluorescence (red) and an overlay of the two (yellow) in COS7 cells transfected with a plasmid encoding an ER-localized ERdj5-mCherry (Pearson’s coefficient of BiP/PDI1A-mCherry co-localization, overlap coefficient r = 0.82, Pearson’s coefficient r = 0.73). The purple Hoechst stains the nucleus. The size bar is 20 μm. **(B)** Trace of time-dependent changes in the normalized intensity of ERdj5-mCherry after photobleaching a small patch of transfected COS7 cell volume. The green trace is of an untreated sample and the orange of cells exposed to thapsigargin 10 minutes before data acquisition. The mean ± SD of the half-time to recovery of the two samples is provided. **(C)** Donor (GFP) fluorescent lifetime of CRT-GFP fusion protein transfected alongside ERdj5-mCherry, as a FRET acceptor. Where indicated the sample was treated with thapsigargin. **(D)** Donor (GFP) fluorescent lifetime of CRT-GFP fusion protein transfected alongside ER-localized mCherry, as a control FRET acceptor or fERdj5-mCherry. **(E)** Donor (GFP) fluorescent lifetime of a GFP-mCherry fusion protein setting the upper limit for FRET. The mean ± SD (vertical white lines) lifetime noted on the histograms. CRT, calreticulin; ER, endoplasmic reticulum; FRET, fluorescence resonance energy transfer; PDI1A, protein disulfide isomerase 1A; SD, standard deviation.

Next, we turned to ERdj5, a PDI family member that has been reported to play an important role in disulfide bond reduction in the ER [4,5,18]. As expected, ERdj5 tagged at its C-terminus with mCherry localized to the ER of transfected COS7 cells (Figure 6A). However, unlike PDI1A (its oxidizing counterpart), mobility of ERdj5-mCherry remained unaffected by ER calcium depletion (Figure 6B). Furthermore, ER calcium depletion did not promote an interaction between ERdj5-mCherry and CRT-GFP, as the FRET signal in cells co-expressing the proteins was unaltered by treatment with thapsigargin (Figure 6C-E).

**Figure 7:**
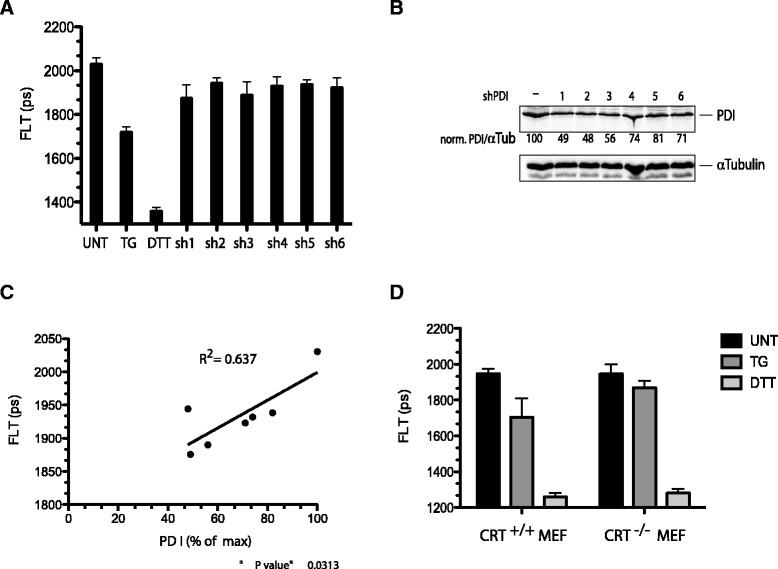
**PDI1A depletion correlates with a more reduced ER. (A)** Bar diagram of fluorescent lifetime (FLT) of roGFPiE in HEK 293 T cells co-transfected with different shRNAs directed to PDI1A and an empty shRNA vector control. FLT of untreated (UNT) thapsigargin (TG) and DTT treated cells is included as a reference. **(B)** Immunoblot of endogenous PDI1A in the cells above, with tubulin serving as a loading control. The PDI1A to tubulin ratio (set to 100 in the vector control sample) is provided under each sample. **(C)** Scatter diagram of correlation of FLT to PDI1A expression in the samples above (R^2^ = 0.637, *P* value = 0.013, n = 7). **(D)** Bar diagram of fluorescent lifetime (FLT) of roGFPiE in wild type mouse embryonic fibroblasts (CRT^+/+^ MEF) and in mouse embryonic fibroblasts lacking calreticulin (CRT^−/−^ MEF) [29]. Where indicated, the cells were exposed to thapsigargin (TG, 0.8 μM for five minutes) or DTT (2 mM, five minutes). Shown is the mean ± SD of FLT (n = 46). (**P <0.001, one way ANOVA for the difference in change in FLT upon TG exposure between the +/+ and −/− cells). ANOVA, analysis of variance; DTT, dithiotreitol; ER, endoplasmic reticulum; PDI1A, protein disulfide isomerase; SD, standard deviation.

The contrasting behavior of the oxidizing and reducing prototypical PDI family members, suggested a role for selective partial immobilization/inactivation of PDI1A in the altered redox poise of the calcium depleted ER. PDI1A is an abundant and long-lived protein; nonetheless, it was possible to bring about a partial transient decrease of its level by shRNA transfection of HEK 293 T cells. The effects of knockdown on ER redox were modest compared with those of thapsigargin treatment (Figure 7A,B). However, the reductive shift, as measured by decreased fluorescence lifetime of ER-localized roGFPiE, correlated well with the extent of PDI1A knockdown across the six different shRNA constructs used (Figure 7C), consistent with a measureable effect of attenuated PDI1A availability on ER redox poise.

Genetic evidence for the importance of CRT to the reductive shift in the calcium-depleted ER was provided by comparison of roGFPiE fluorescent lifetime in wild type and CRT-deficient mouse embryonic fibroblasts. The absence of CRT had no effect on the basal redox state of the probe or on its redox state in cells exposed to DTT. However, the reductive shift observed upon exposure of the wild type cells to the ER calcium depleting agent thapsigargin (reflected in a shortening of lifetime from 1,947 ± 28 to 1,701 ± 107 ps), was blunted in the CRT-deficient cells (shortening of lifetime from 1,946 ± 53 to 1,869 ± 38 ps) (Figure 7D) indicating a non-redundant role for CRT in promoting the reductive shift in the calcium depleted ER.

## Discussion

The reductive shift in ER protein thiol redox poise that follows lumenal calcium depletion is shown here to correlate with a kinetic defect in the rate of recovery of disulfides following a reductive pulse and its washout. However, the core enzymatic machinery that produces disulfides in the ER and conveys them to reduced protein substrates is unresponsive to calcium *in vitro. In vivo*, however, the mobility of the prototypical oxidizing PDI, PDI1A, is lowered by calcium depletion and this correlates with the formation of a calcium-suppressible complex between PDI1A and the ER chaperone CRT, both *in vitro* and *in vivo*.

**Figure 8:**
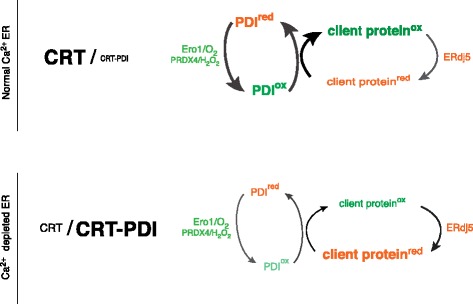
**Schematic of the effects of lumenal calcium depletion on availability of PDI1A to fuel client protein oxidation in the ER.** In the calcium replete ER, PDI1A is free to engage in oxidative protein folding by shuttling electrons from reduced client protein (client protein^red^) to terminal acceptors (via ER oxidases, such as ERO1 and PRDX4) and oxidation (to client protein^ox^) competes favorably with reduction (exemplified by the action of ERdj5). In the calcium depleted ER, PDI1A is sequestered in a low mobility complex with the abundant chaperone calreticulin, compromising its availability to power the oxidative limb of the ER. The reductive limb, mediated by ERdj5, is unaffected by calcium depletion. The net result is a shift in the balance of the calcium-depleted ER towards a more reduced poise. ER, endoplasmic reticulum; PDI1A, protein disulfide isomerase 1A.

While our findings fall short of establishing causality between impaired mobility of PDI1A and the oxidative defect in the calcium-depleted ER lumen, the selectivity of the immobilization for the oxidizing PDI family member, reflected in the sparing of ERdj5 mobility, supports such a link. Variation in calcium concentrations within the physiological range regulate the formation of a previously-observed complex of PDI1A and CRT [13], suggesting that this complex (or similar complexes that may form in parallel), account for the reduced mobility of the oxidizing PDI1A and, therefore, a selectively attenuated turnover of the oxidizing limb of the ER lumen (Figure 8). The reductive shift noted upon ER calcium depletion is significantly attenuated but not completely lost in cells lacking CRT, indicating the existence of other contributory factors.

CRT is well suited to serve as a calcium sensor in the interaction with PDI1A, as it has been noted to change its tertiary structure in response to changes in calcium concentration, with increased exposure of hydrophobic surface as calcium concentration decreases [14]. PDI1A and CRT are both abundant ER proteins and their C-termini are rich in acidic residues that could mediate calcium responsiveness of complex formation. However, deletion of the C-terminal regions (that are not essential to structural and functional integrity) proved insufficient to abolish the calcium depletion-mediated interaction, frustrating our efforts at gross deletional mapping of the calcium-dependent interaction between them.

Association with CRT does not inactivate PDI1A *in vitro*, and neither complex formation, nor *in vivo* mobility exhibit measureable specificity for the oxidized form of PDI1A. Rather, our findings fit best with non-selective sequestration of both reduced and oxidized PDI1A in lower mobility complex(es) in the calcium depleted ER. The FRAP traces of PDI1A do not resolve into a simple model of only two pools (free and CRT-bound), thus the PDI1A-CRT complex may be heterogeneous or PDI1A may interact with other proteins too, forming an ensemble of complexes that diffuse slowly in the calcium-depleted ER. Indeed, the 2.5 fold decrease in PDI1A upon calcium depletion fits with a complex larger than merely a PDI1A-CRT dimer. Unfortunately, instability of the PDI1A-CRT complex has frustrated our efforts at isolation and biochemical characterization. Despite these limitations to our understanding, the mechanism(s) promoting a reductive shift in the calcium depleted ER are defined with considerable specificity. That a reductive shift is not observed in conditions associated with markedly elevated levels of unfolded protein stress in the ER (for example, in tunicamycin-treated cells [9]) argues that promiscuous effects of calcium depletion on the handling of secreted proteins are unlikely to contribute. The evidence for specificity is further buttressed by the significant effacement of the reductive shift in cells lacking CRT.

What might be the physiological significance of the reductive shift of the calcium-depleted ER? It is best to begin by acknowledging the possibility that redox effects may not be under positive selection but rather reflect a consequence of a role for PDI1A in maintaining CRT’s solubility through physiological fluctuations in [Ca^2+^]_ER_. However, it is also intriguing to consider this question in the context of physiological circumstances associated with calcium excursions into the range that effect PDI1A-CRT association and ER reduction. Such excursions are noted in secretory cells responding to secretagogues and are associated with physiological activation of the unfolded protein response [19]. The reductive shift in the ER may contribute to such activation, transiently inhibiting protein synthesis, through rapidly reversible protein kinase RNA-like endoplasmic reticulum kinase (PERK)-mediated eIF2 phosphorylation [20]. Transient inhibition of energetically-costly protein synthesis may be especially important in insulin-producing beta cells, in which coupling of insulin secretion to blood glucose requires high levels of intracellular ATP [21]. Furthermore, coupling secretory stimuli (which are transient) to periodic activation of UPR target genes would have enduring effects on ER size and on the long-term capacity to produce secreted products. The evolution of an ER that is more reducing under conditions of ER calcium store depletion would promote fitness by coupling the action of calcium-mobilizing secretagogues to a UPR-mediated transient attenuation of protein synthesis followed by long-term growth of the ER.

Transient reductive departures from the normally oxidizing poise of the ER may provide other benefits to cells. Shifting the balance from the oxidizing PDI1A to the reducing ERdj5 is predicted to favor degradation of disulfide bonded misfolded proteins [4]. Thus, transient cycles of reduction may be helpful in periodically purging the ER of recalcitrant misfolded proteins, while maintaining ER redox at levels optimized for oxidative folding most of the time. Coupling such reductive shifts to PERK-mediated attenuation of protein synthesis would lessen their impact on oxidative folding of newly-synthesized proteins, while promoting the degradation of refuse.

A functional link between ER redox and calcium homeostasis is suggested by the phenotype of mice lacking ERO1 genes. Cells explanted from such animals have no steady state defect in ER thiol redox poise, but recover sluggishly from reductive excursions [9]. The mice are superficially indistinguishable from wild type; however, calcium handling by cardiomyocytes is grossly impaired, supporting a role for rapid kinetics of disulfide recovery in ER calcium release and re-uptake [22].

Calcium handling by the ER is subject to redox regulation. Levels of ERO1 activity modulate calcium fluxes in and out of the ER [23]. Subtype 1 of the IP3 receptor, which conducts lumenal calcium to the cytosol in response to activation of membrane receptors, is inhibited by the formation of a mixed disulfide between cysteine residues on its third lumenal loop and reduced ERp44 [24], a reducing PDI family member. By contrast, the broadly-expressed calcium reuptake pump, SERCA2b is inhibited by the formation of a disulfide with the oxidizing PDI family member ERp57 [25]. As disulfide exchange between PDI family members is pervasive, these observations suggest that a more reduced ER poise would favor re-uptake of calcium into the ER and disfavor its release. It follows that immobilization of the oxidizing PDI1A, which can transfer disulfides to ERp57 [26], and continued mobility of the reducing ERdj5 would favor restoration of calcium stores to the calcium-depleted ER closing a homeostatic loop.

## Conclusions

The reductive shift in ER redox poise, observed at physiological ER calcium store depletion, is an unusual exception to a landscape dominated by stability of ER redox balance. Direct calcium responsiveness of the core machinery for oxidizing ER client protein thiols has been dismissed by our studies. However a remarkable correlation between lumenal calcium depletion and lowered mobility of the oxidizing PDI1A in the ER lumen was observed. This, in turn, is plausibly explained by formation of a calcium responsive complex between PDI1A and the abundant ER chaperone CRT with the effect of sequestering a key component of the oxidizing limb of the ER (Figure 8). The consequent attenuation of client protein oxidation accompanies physiological signals that specify secretion, thus delineating a hitherto unanticipated link between secretagogue action and protein folding homeostasis in the ER.

## Methods

### Plasmid construction

Additional file 2: Table S1 lists the plasmids used, their lab names, description, published reference and a notation of their appearance in the figures.

### Protein purification and *in vitro* enzymatic assays

Human PDI (PDIA1 18–508), mouse ERO1L and roGFPiE were expressed in the *E. coli* BL21 (DE3) strain and purified with Ni-NTA affinity chromatography or glutathione affinity chromatography as previously described [9,27]. Time-dependent changes in redox of roGFPiE, in the presence of ERO1 and PDI1, were measured as described [9,28].

### Transfections, immunoblotting, immunofluorescence and cell culture

HEK 293 T, COS7 and AR42j cells were obtained from the (ATCC, Teddington, Middlesex, UK). Mouse embryonic fibroblasts lacking CRT and their wild type counterparts were a gift of Marek Michalak (University of Alberta, Alberta, Canada) [29]. Cells were cultured in DMEM (SIgma, Gillingham, Dorset, UK), supplemented with 10% fetal calf serum. Transfections were performed using the Neon Transfection System (Invitrogen, Paisley, UK). For shRNA knock down, stably transfected HEK293T cells were selected in puromycin (2 μg/ml). Cells from confluent 100-mm dishes were washed in phosphate-buffered saline (PBS), lysed in 0.5% Triton X-100, 150 mM NaCl, 20 mM Hepes, pH 7.4, and protease inhibitors. Proteins were resolved by 12% SDS-PAGE and blotted with a mouse monoclonal anti-PDI (1D3 Assay Designs, Exeter, UK).

C/EBP Homologous Protein (CHOP) immune-reactivity was detected by immunofluorescence as described [30]. To localize PDI-mCherry and ERdj5-mCherry the proteins were visualized alongside BiP in 2% paraformaldehyde fixed cells using a chicken polyclonal antibody raised in hen against full-length bacterially-expressed hamster BiP [9], anti-chicken DyLight488 conjugated immunoglobulin G (IgG) was used as a secondary antibody (Jackson ImmunoResearch Laboratories, West Grove, PA, USA).

### Protein purification and enzymatic assays

For *in vitro* assays, human PDI (PDIA1 18–508, a gift of Colin Thorpe, University of Delaware, Newark, DE, USA), mouse Ero1α and roGFPiE were expressed in *E. coli* BL21 (D3) strain, purified with Ni-NTA affinity chromatography, and dialyzed into the reaction buffer, and reduced by incubation with 20 mM of DTT, and then buffer exchanged on a PD-10 gel filtration column (GE Healthcare, Little Chalfont, Buckinghamshire, UK) [27]. For kinetic assay, reduced PDI (0 to 150 μM) was equilibrated in degased 100 mM HEPES pH 7.4, 150 mM NaCl buffer containing an excess of a combination of reduced and oxidized glutathione (4 mM, Sigma) for one hour at room temperature, then added simultaneously to all the samples in the experiment containing 2 μM reduced roGFPiE. For the assessment of Ero1 activity the enzyme (0 to 15 μM) was added to reduced PDI (30 μM) and roGFPiE (2 μM). The ratio of fluorescence emission at 520 nm following sequential excitation at 395 and 470 nm was measured using EnSpire® Multimode Plate Readers (PerkinElmer, Waltham, MA, USA).

To assess the calcium dependence of PDI-mCherry/CRT-GFP interaction *in vitro* the proteins were equilibrated in 50 mM HEPES pH = 7.2, 100 mM KCl buffer containing 2 mM CaCl_2_, then increasing amounts of ethyleneglycoltetraacetic acid (EGTA) were introduced to yield a range of free Ca^2+^ concentrations (8 to 2,000 μM, calculated using Maxchelator, Stanford University, Palo Alto, CA, USA). FRET signal was detected measuring the ratio between the emission at 630 nm and 520 nm, both excited at 485 nm.

### FLIM and intensity-based confocal microscopy and image analysis

A Zeiss 510 Meta laser scanning confocal system with Plan-Apochromat ×63 oil immersion lens (NA = 1.6) was used to acquire intensity based fluorescence microscopy images of cells fixed with 2% paraformaldehyde PBS. Images were analyzed using ImageJ with JACoP (National Institutes of Health, Bethesda, MD, USA) to assess colocalization.

FLIM experiments were performed on a modified version of a previously-described laser scanning multiparametric imaging system [31], coupled to a microscope incubator, maintaining standard tissue culture conditions (Okolab, Pozzuoli, NA, Italy), using a pulsed (sub 10 ps, 20 MHz) supercontinuum (430 to 2,000 nm) light source (SC 450, Fianium Ltd., Southampton, UK). Desired excitation wavelength (typically 470 nm for FLIM of GFP variants and 440 nm for D1ER cameleon FRET) was tuned using an acousto-optic tunable filter (AA Opto-electronic AOTFnC-VIS, Orsay, France). Desired emission was collected using 510/42 and 470 to 490 nm bandpass filters for GFP variants and D1ER cameleon accordingly and detected by a fast photomultiplier tube (PMC-100, Becker & Hickl GmbH, Berlin, Germany). Lifetimes for each pixel were recorded using time correlated single photon counting (TCSPC) circuitry (SPC-830, Becker & Hickl GmbH), keeping count rates below 1% of the laser repetition rate to prevent pulse pile-up. Images were acquired over 20 to 60 seconds, with a typical flow rate of 5 × 10^4^ photons sec^−1^_,_ while detectors speed saturation (pile up effect) is not observed below 10^6^ photons sec^−1^ in this instrument. The data were processed using SPCImage (Becker & Hickl GmbH) fitting the time correlated photon count data obtained for each pixel of the image to a mono-exponential decay function, yielding a value for lifetime on the pico-second scale.

After filtering out autofluorescence (by excluding pixels with a fluorescence lifetime that is out of range of the roGFP probes, that is, longer than 2,800 ps), mean fluorescence lifetime of single cells was established. The value obtained represented the redox as sensed by roGFPiE, FRET efficiency of D1ER cameleon (inversely proportional to Ca^2+^ concentration) or of GFP/mCherry. Each data point is constituted by the average and SD of measurements from at least 10 cells. For GFP/mCherry *in vivo* FLIM FRET, cells with sufficient expression of mCherry were selected based on the signal obtained from excitation at 550 nm and 612/35 emission. FRET efficiency was calculated using the equation: E_FRET_ = 1 ‐ τ_DA_/ τ_D_, where τ_D_ and τ_DA_ are the lifetime values of the FRET donor in the absence or presence of an acceptor, respectively. For calculations of interacting fractions of CRT-GFP, lifetime of GFP-mCherry fusion was assumed to represent 100% interacting fraction [32].

For FRAP analysis of mCherry and GFP fused proteins a series of 60 to 140 images with two second intervals were acquired. Bleaching in a selected region of interest (ROI) was introduced at image 10, using 100% laser power (488 nm for GFP fused proteins and 514/543 nm for mCherry fused proteins, 100 to 300 iterations). Single recovery halftime (t_1/2_) was calculated using the FRAP macro of ZEN software (Carl Zeiss Microimaging, Oberkochen, Germany).

### Calcium imaging

The FRET based, ER-optimized D1ER cameleon [16], was used for ER calcium imaging by FLIM (exploiting fluorescence lifetime shortening effect of FRET on the emission of the cyan fluorescent protein segment of D1ER cameleon). Concentration of ER calcium was calculated from the fluorescence lifetime value based on the equation: [Ca^2 +^] = (k_d_ * (τ_max_–τ)/(τ ‐ τ_min_))^1/h^, where k_d_ is the apparent dissociation constant of the probe and h is Hill’s coefficient (197 μM and 0.87, respectively, measured i*n vivo* [33]), τ_max_ and τ_min_ are the highest and the lowest values of the probes’ lifetime measured in permeablized cells exposed to buffers with 0 or saturating calcium, respectively (full method will be published separately).

## Additional files

Additional file 1: Figure S1. Tunicamycin-induced activation of a UPR marker gene. **Figure S2.** Calcium depletion-mediated attenuation of PDI1A mobility is also observed following reduction of its active sites. **Figure S3.** PDI1A attenuates calcium chelation-induced aggregation of calreticulin. **Figure S4.** Calcium depletion-mediated association of CRT and PDI1A is also observed under reducing conditions. **Figure S5.** Calcium-dependent association with CRT does not affect PDI1A’s ability to promote disulfide bond formation. Additional file 2: Table S1. Listing the plasmids used in this study, their unique lab identification number, name, description, published reference, and a notation of their first appearance in the figures.
