# Supplementary material for: Retarded PDI diffusion and a reductive shift in poise of the calcium depleted endoplasmic reticulum
Source: BMC Biol. 2015 Jan 10;13:2. doi: 10.1186/s12915-014-0112-2 (PMC4316587; doi:10.1186/s12915-014-0112-2)
Supplement: Additional file 1: Figure S1. — Tunicamycin-induced activation of a UPR marker gene. Figure S2. Calcium depletion-mediated attenuation of PDI1A mobility is also observed following reduction of its active sites. Figure S3. PDI1A attenuates calcium chelation-induced aggregation of calreticulin. Figure S4. Calcium depletion-mediated association of CRT and PDI1A is also observed under reducing conditions. Figure S5. Calcium-dependent association with CRT does not affect PDI1A’s ability to promote disulfide bond formation. [file 12915_2014_112_MOESM1_ESM.pdf]

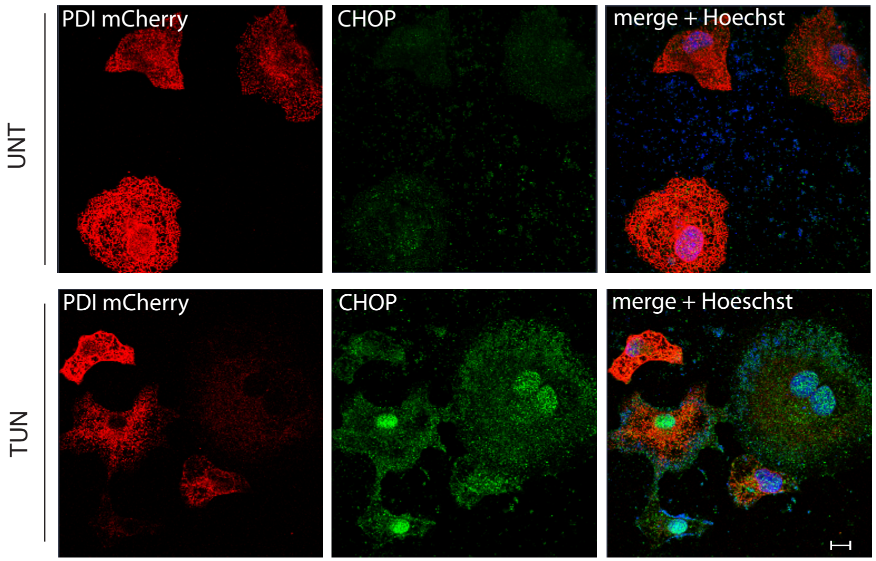

Figure S1. Tunicamycin-induced activation of a UPR marker gene. Photomicrographs of untreated and tunicamycin-treated PDI1A-mCherry-expressing COS7 cells, revealing the mCherry fluorescent signal (red) and the immunostaining of the ER stress marker CHOP (green). The purple Hoechst stains the nucleus. The size bar is 20 µm. Note the induction of CHOP by tunicamycin in COS7 cells treated as in Figure 2G.

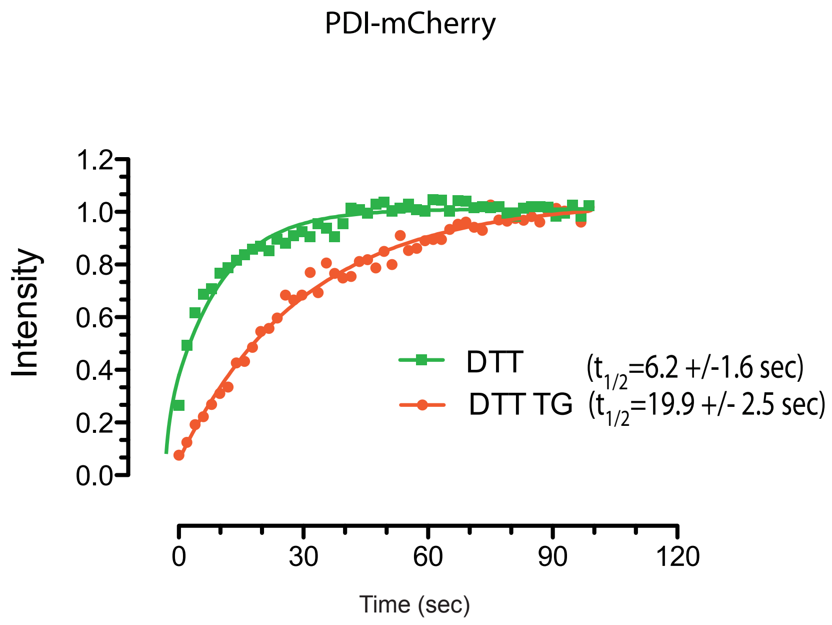

Figure S2. Calcium depletion-mediated attenuation of PDI1A mobility is also observed following reduction of its active sites. Trace of time-dependent recovery in the normalized intensity of PDI1A-mCherry after photobleaching a small patch of transfected COS7 cell volume. Both samples were exposed to the reducing agent dithiothreitol (DTT, 2 mM), 10 minutes before the FRAP experiment. The sample with the red trace was coincidentally exposed to thapsigargin. The thapsigargin-mediated delay in fluorescent recovery noted in Figure 2C is also observed in DTT-treated samples.

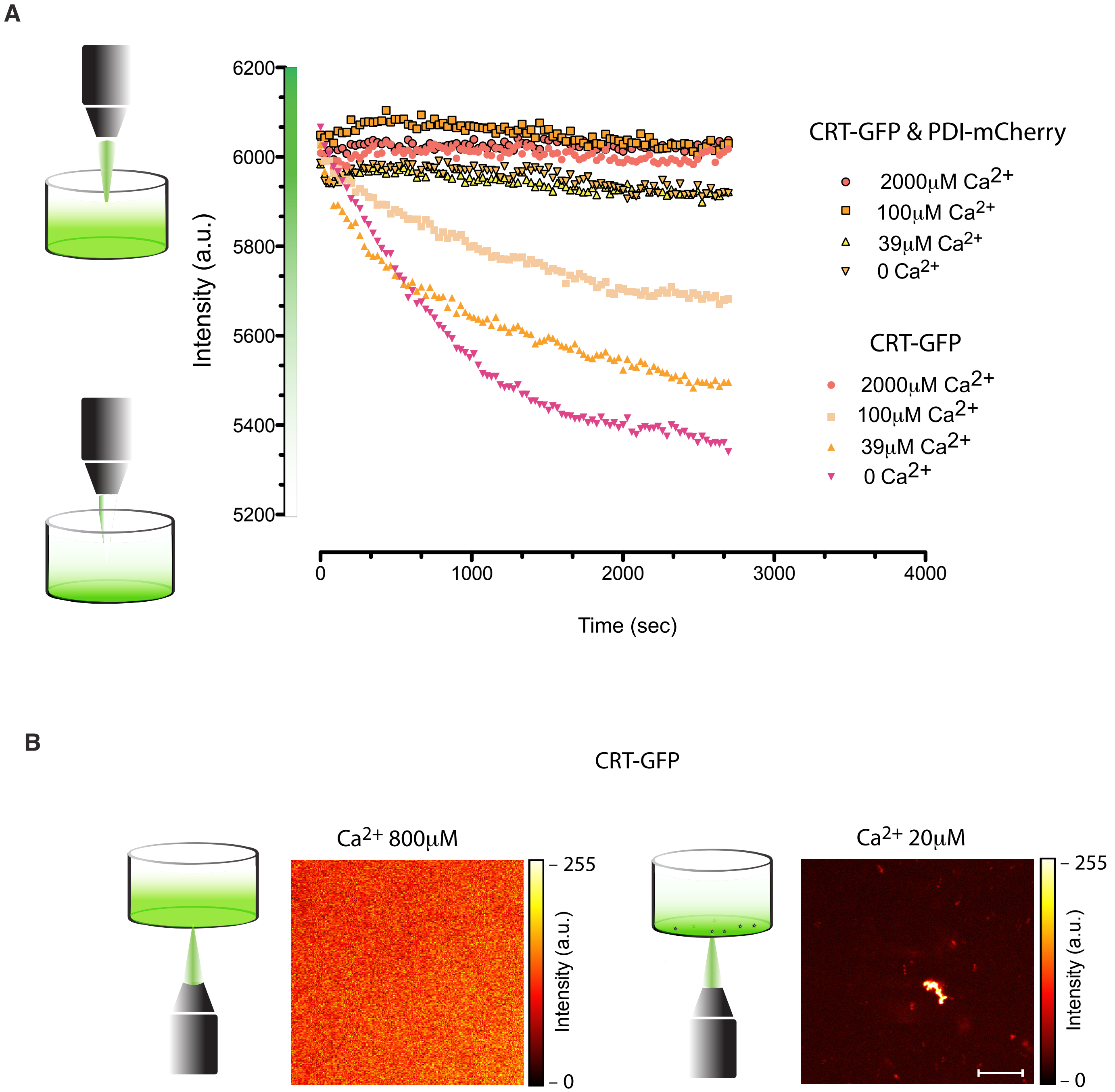

Figure S3. PDI1A attenuates calcium chelation induced aggregation of calreticulin. (A) Trace of time-dependent change in fluorescence intensity of solutions of calreticulin-GFP fusion protein (2 μM) viewed from above, at the indicated calcium concentrations in the presence and absence of PDI1A (5 μM). Disappearance of fluorescent signal correlates with aggregation of the calreticulin-GFP fusion protein. (B) Representative fluorescent photomicrographs of solutions of a calreticulin-GFP fusion protein (2 μM) at the indicated calcium concentrations. Note the diffuse fluorescent signal in the sample maintained at physiological concentrations of calcium (800 μM) and the patchy distribution of intense fluorescence in the sample maintained in low calcium (20 μM). The size bar is 20 μm.

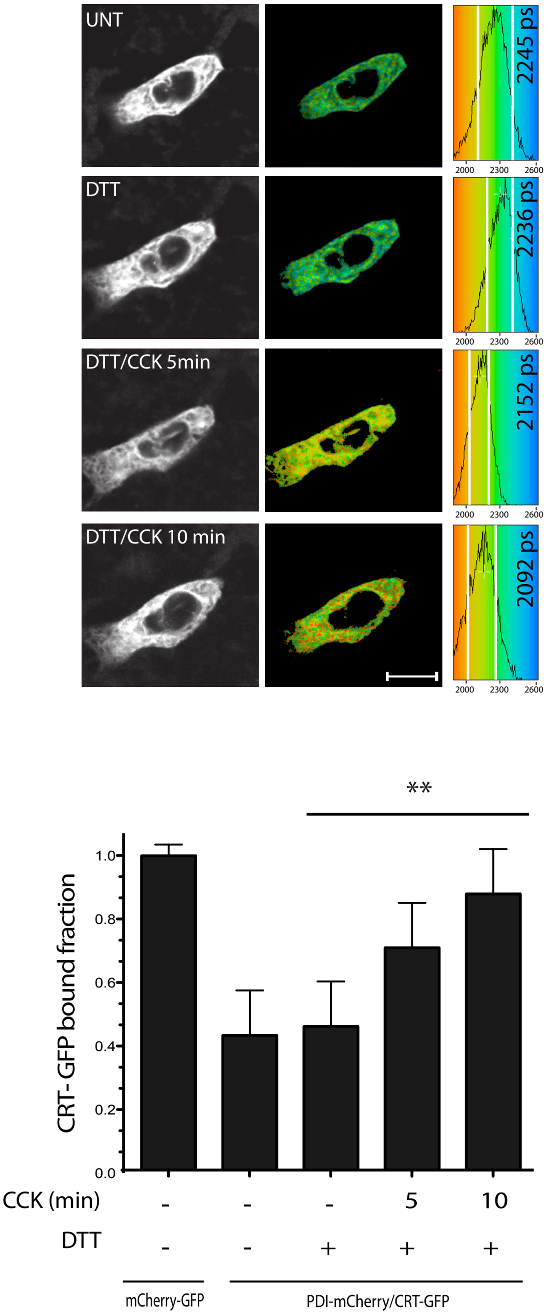

Figure S4. Calcium depletion-mediated association of CRT and PDI1A is also observed under reducing conditions. (A) Donor (GFP) fluorescent lifetime of CRT-GFP fusion protein transfected alongside PDI1A-mCherry, as a FRET acceptor into AR42J cells. Samples were treated with DTT (2 mM), CCK (10 µM) or both. The size bar is 20 µm. (B) Quantification of complex formation between CRT-GFP and PDI1A-mCherry in the experiments shown in "A", above.

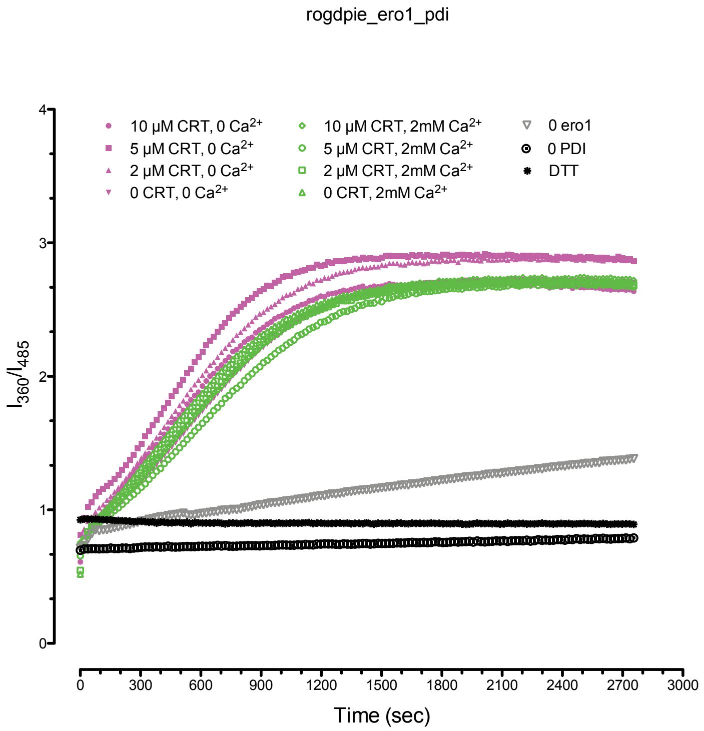

Figure S5. Calcium-dependent association with CRT does not affect PDI1A's ability to promote disulfide bond formation. Shown are traces of time-dependent change in roGFPiE oxidation in vitro (measured by the ratio of excitation intensity at 470 nm and 395 nm) in samples of reduced probe exposed to reduced PDI1A (5 µM) and ERO1 (0.5 µM) in absence or presence of CRT at the indicated concentration of calcium. Note that PDI1A is able to oxidize roGFPiE in solutions lacking calcium.
