# Supplementary material for: Retarded PDI diffusion and a reductive shift in poise of the calcium depleted endoplasmic reticulum
Source: BMC Biol. 2015 Jan 10;13:2. doi: 10.1186/s12915-014-0112-2 (PMC4316587; doi:10.1186/s12915-014-0112-2)
Supplement: Additional file 2: Table S1. — Listing the plasmids used in this study, their unique lab identification number, name, description, published reference, and a notation of their first appearance in the figures. [file 12915_2014_112_MOESM2_ESM.pdf]

Unique ID	Plasmid Name	Description	Reference	First appearance	Label in figure
527	FLAGM1_roGFP_iE_pCDNA3	Mammalian expression, ER-localised roGFPiE	PMID: 23589496	1A	ERroGFPiE
355	roGFP_iE_PQE30	Bacterial expression roGFPiE	PMID: 18652491	1B	roGFPiE
233	hPDI(18-508)pTrcHis-A	Bacterial expression of human PDI1A	PMID: 21145486	1B	PDI
221	mERO1a(43-464).pGS	Bacterial expression of mouse ERO1 alpha	PMID: 20442408	1D	ERO1
887	hPDI_WT_mCherry_KDEL-N3	Mammalian expression of a human PDI1A-mCherry_KDEL fusion protein	This paper	2B	PDI-mCherry
888	pFLAG_mCherry_KDEL_CMV1	Mammalian expression of FLAG M1-tagged ER-localised mCherry-KDEL	This paper	2E	ERmCherry
1032	rCRT_18_405_HA_EGFP_KDEL_V2_pGEX_TEV	Bacterial expression rat calreticuin fused to EGFP	This paper	3A	CRT-GFP
1033	hPDI_18-505_mCherry_pGV67	Bacterial expression PDI1A-mCherry fusion protein	This paper	3A	PDI-mCherry
906	mCherry_TEV_PQE10	Bacterial expression of mCherry	This paper	3B	mCherry
700	D1ER_Cameleon_pCDNA3	Mammalian expression of ER-targeted D1 Cameleon	PMID: 15585581	4A	D1ER Cameleon
880	pmCherry_N3	Mamalian expression of mCherry	Clonetech	4B	mCherry
20	pEGFP-C1	Mamalian expression of EGFP	Clontech	4B	GFP
1401	mCherry17aaGFP	Mammalian expression of an EGFP-mCherry fusion protein	PMID: 18770849	4C	GFP-mCherry fusion
699	CRT_GFP	Mammalian expression of GFP tagged rat calreticulin	PMID: 16617114	4D	CRT-GFP
1280	mERdj5_1-789_mCherry_KDEL_N3	Mammalian expression mouse ERdj5-mCherry fusion protein	This paper	6A	ERdj5-mCherry
1208	hPDIshRNA_plKO1_296736	Sigma mission shRNA against human PDI clone1	Sigma mission shRNA	7A	sh1
1209	hPDIshRNA_plKO1_290651	Sigma mission shRNA against human PDI clone2	Sigma mission shRNA	7A	sh2
1210	hPDIshRNA_plKO1_296675	Sigma mission shRNA against human PDI clone3	Sigma mission shRNA	7A	sh3
1211	hPDIshRNA_plKO1_49194	Sigma mission shRNA against human PDI clone4	Sigma mission shRNA	7A	sh4
1212	hPDIshRNA_plKO1_49197	Sigma mission shRNA against human PDI clone5	Sigma mission shRNA	7A	sh5

Table S1. Table listing the plasmids used in this study, their unique lab identification number, name, description, published reference and a notation of their first appearance in the figures.
